# Immune Checkpoint Inhibitors as a Treatment Option for Bladder Cancer: Current Evidence

**DOI:** 10.7759/cureus.40031

**Published:** 2023-06-06

**Authors:** Tobechukwu J Okobi, Trinitas Oserefuamen Uhomoibhi, Darlington E Akahara, Victor A Odoma, Ibilola A Sanusi, Okelue E Okobi, Ifiok Umana, Emeka Okobi, Chinwe C Okonkwo, Nkechinyere M Harry

**Affiliations:** 1 Internal Medicine, Bronx Care Health System, New York, USA; 2 Internal Medicine, Georgetown University, Washington, D.C., USA; 3 Internal Medicine, University of the District of Columbia, Washington, D.C., USA; 4 Medicine, Windsor University School of Medicine, Cayon, KNA; 5 Cardiology/Oncology, IU Health, Bloomington, USA; 6 Internal Medicine, University of Texas at Houston, Houston, USA; 7 Family Medicine, Medficient Health Systems, Laurel, USA; 8 Family Medicine, Lakeside Medical Center, Belle Glade, USA; 9 Urology, Jos University Teaching Hospital, Jos, NGA; 10 Dentistry, Ahmadu Bello University Teaching Hospital Zaria, Abuja, NGA; 11 Family Medicine, Caribbean Medical University School of Medicine, Willemstad, CUW; 12 Internal Medicine, National Pirogov Memorial University, Vinnitsa, UKR

**Keywords:** biomarkers, immune check point inhibitors, treatment, bladder cancer, immunotherapy

## Abstract

Bladder cancer is a prevalent disease, and treatment options for advanced bladder cancer remain limited. However, immune checkpoint inhibitors (ICIs) targeting cytotoxic T-lymphocyte-associated antigen-4 (CTLA-4) and programmed cell death-1 (PD-1) have shown promise in treating bladder cancer. These drugs work by blocking receptors and ligands, disrupting signaling, and allowing T cells to recognize and attack cancer cells. ICIs have been found to be effective in treating bladder cancer, especially in cases of metastatic urothelial carcinoma (UC) that have progressed after chemotherapy. Furthermore, combination therapy with ICIs and chemotherapy or radiation therapy has shown promise in treating bladder cancer. While there are challenges associated with ICIs, including adverse effects, immune-related adverse events, and lack of efficacy in some patients, they remain a promising option for bladder cancer treatment, especially in cases where other treatment options have failed. This review paper focuses on the current role, challenges, and future trends of immunotherapy in the management of bladder cancer.

## Introduction and background

Bladder cancer is a significant public health concern and the leading cause of worldwide cancer-related mortality. It is the tenth most common cancer worldwide and the second most common urinary tract malignancy globally, accounting for 549,000 new cases and approximately 200,000 deaths annually [1-3]. The incidence and mortality rates vary widely across different regions of the world, with the highest incidence rates reported in Western countries, including North America and Europe. In the US, it is the sixth most common cancer diagnosed, representing 3% of all cancers recorded worldwide [2-6]. Over 80,000 new cases with over 16,000 deaths are estimated to occur in 2023. Its 5-year survival rate is approximately 70% for localized cancers, 39.2% for regional, and <10% for distant metastatic cancers [3]. The disease is associated with poorer outcomes, especially with distant spread, so continued efforts to improve management options remain crucial.

Types of bladder cancers

**Figure 1:**
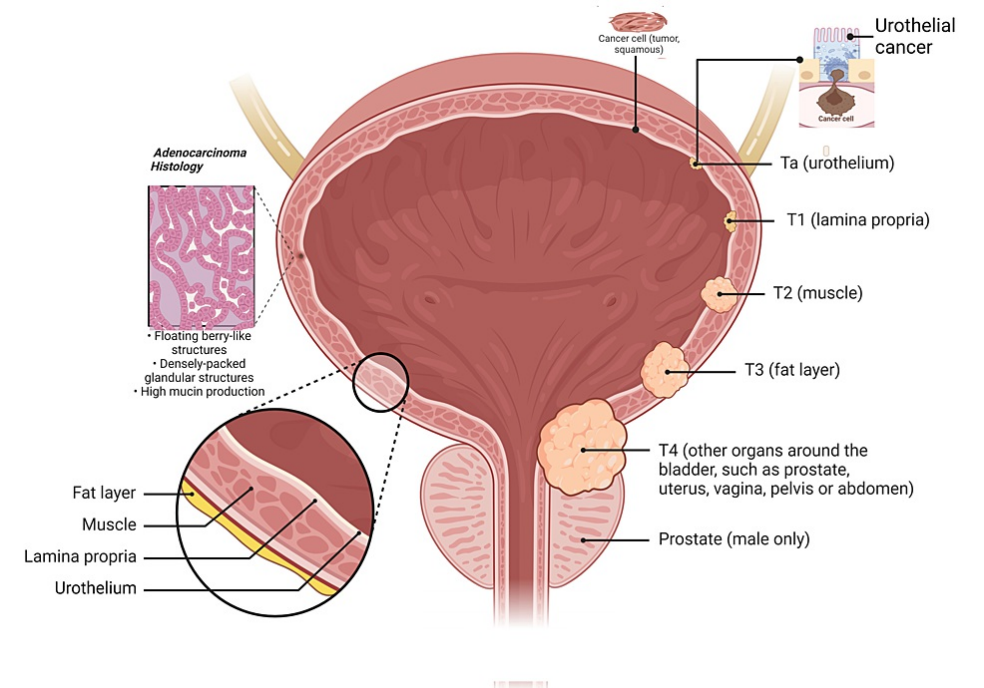
Bladder Cancer Types and Staging This illustration is an original creation of the authors of this manuscript

Bladder cancer is a disease that originates in the cells of the bladder. While the most common type of bladder cancer is urothelial carcinoma (also known as transitional cell carcinoma), there are other less common types as well. Here is a brief overview of some of the less common types of bladder cancer (Figure 1).

Squamous Cell Bladder Cancer

This type of bladder cancer develops from the squamous cells, which are thin, flat cells that may line the bladder in response to chronic irritation or process. Squamous cell bladder cancer accounts for a small percentage of bladder cancers and is often associated with chronic inflammation or condition, such as long-term bladder stones or urinary tract infections.

Adenocarcinoma

Adenocarcinoma of the bladder is a rare type of bladder cancer that develops from the glandular cells in the bladder. These cells typically produce mucus-like substances. Adenocarcinoma may arise from urachal remnants, which are embryonic structures that connect the bladder to the umbilical cord during fetal development.

Sarcoma

Bladder sarcoma is an extremely rare type of bladder cancer that develops in the connective tissues of the bladder, such as muscle, fat, or blood vessels. Sarcomas can be aggressive and tend to grow rapidly, but they account for a very small percentage of bladder cancers.

Small Cell Bladder Cancer

Small cell bladder cancer is an uncommon and aggressive type of bladder cancer that is histologically similar to small cell lung cancer. It is characterized by small, tightly packed cells and is often associated with a smoking history. Small cell bladder cancer tends to grow quickly and may metastasize (spread) to other body parts early in its course.

Apart from these less common types, there are also other rare variants of bladder cancer, including squamous cell carcinoma with glandular differentiation, micropapillary carcinoma, plasmacytoid carcinoma, and neuroendocrine tumors. It's important to note that the treatment and prognosis for these less common types of bladder cancer may differ from those for urothelial carcinoma, and specialized medical attention is necessary for accurate diagnosis and appropriate management [2,7-8].

Immunotherapy in cancer management

Multiple modalities of immunotherapy have been employed in the management of cancer. These encompass immune checkpoint inhibitors, which are pharmaceutical agents that inhibit immune checkpoints. Additionally, T-cell transfer therapy has emerged as a treatment modality that fortifies the intrinsic capacity of T cells to combat cancer. This approach is sometimes referred to as adoptive cell therapy, adoptive immunotherapy, or immune cell therapy. Another avenue involves the utilization of monoclonal antibodies, which are artificially generated immune system proteins meticulously designed to bind to specific targets on malignant cells. Treatment vaccines, on the other hand, augment the immune system's anticancer response by eliciting an enhanced immune reaction against cancer cells. Lastly, immune system modulators have been developed to heighten the body's immune response against cancerous entities.

Immune checkpoint inhibitors (ICIs) are a type of cancer immunotherapy that helps to enhance the body's natural immune response against cancer cells. They work by targeting specific proteins known as immune checkpoints that are present in immune cells and cancer cells. In a healthy immune system, immune checkpoints play a crucial role in preventing excessive immune responses and maintaining self-tolerance. However, cancer cells can hijack these checkpoints to evade the immune system and continue growing unchecked. Immune checkpoint inhibitors block the interaction between immune checkpoints, such as programmed cell death protein 1 (PD-1) and cytotoxic T-lymphocyte-associated antigen 4 (CTLA-4), and their corresponding ligands on cancer cells. This blockade releases the "brakes" on the immune system, allowing immune cells to recognize and attack cancer cells more effectively. By unleashing the immune system's full potential, immune checkpoint inhibitors can promote long-lasting anti-tumor responses. They have shown significant success in treating various types of cancers, including melanoma, lung cancer, kidney cancer, bladder cancer, and others. However, the response to immune checkpoint inhibitors can vary among individuals and cancer types.

It's important to note that immune checkpoint inhibitors can also lead to immune-related side effects, as the enhanced immune response may target normal cells and tissues. These side effects can affect various organs and systems, and prompt monitoring and management by healthcare professionals. Overall, immune checkpoint inhibitors have revolutionized cancer therapy and have become a valuable addition to the treatment options available, offering new hope for patients with certain types of cancer. A notable example is pembrolizumab, a medication that targets the PD-1 checkpoint and is approved for the treatment of various types of cancer, including melanoma, non-small cell lung cancer, head and neck squamous cell carcinoma, and others. Another notable example is nivolumab, which also targets the PD-1 checkpoint and is approved for the treatment of several cancers, including melanoma, non-small cell lung cancer, renal cell carcinoma, and others.

Atezolizumab is also another ICIC that targets the programmed death-ligand 1 (PD-L1) checkpoint and is approved for the treatment of bladder cancer, non-small cell lung cancer, and triple-negative breast cancer, among others. Ipilimumab is also noted to target the CTLA-4 checkpoint and is approved for the treatment of melanoma. Durvalumab, another notable example, targets the PD-L1 checkpoint and is approved for the treatment of bladder cancer, non-small cell lung cancer, and small cell lung cancer. Lastly, there are other ICIs being developed and used in clinical trials for various types of cancer. The specialized use of these medications depends on the type and stage of the cancer being treated, as well as other individual factors [4,9-16].

Over the last decade, immunotherapy has revolutionized the treatment of bladder cancer. The first anti-cancer drug targeting an immune checkpoint was ipilimumab, a CTLA4 blocker approved in the United States in 2011, while nivolumab and pembrolizumab (PD-1 inhibitors) were approved for urothelial carcinoma treatment in 2014 either as a single agent, in combination with other immunotherapy, or with chemotherapy and radiation therapy, with several clinical trials currently ongoing to optimize its use [4-10]. The primary research objective of this manuscript is to highlight some of the evolving roles immune checkpoint inhibitors play in treating bladder cancer. 

Methodology

In our methodology, we first defined our research objective clearly. Next, we developed a search strategy by determining relevant keywords and search terms (Immunotherapy, bladder cancer, treatment, Immune checkpoint inhibitors, biomarkers) that align with our objective. To refine our search, we utilized advanced search operators and filters (such as "AND" and "OR") available in PUBMED. For selecting literature sources, we relied on PubMed, which provides access to a vast collection of scholarly articles, journals, and publications in the field of medicine and healthcare. We specifically focused on primary research studies, systematic reviews, meta-analyses, and other relevant sources. To streamline our search, we limited our search engine to PubMed only.

To screen and select articles, we established specific criteria based on our research objectives. We applied practical screening criteria such as relevance, publication date, and study design to filter out irrelevant studies. Once we had our selected articles, we extracted relevant information and data from them, organizing and synthesizing our findings in a structured manner. We identified common themes, trends, patterns, and key arguments across the literature. To manage our data effectively, we utilized tools such as spreadsheets or reference management software.

Afterwards, we critically evaluated the strengths and weaknesses of the included studies, assessing their credibility, reliability, and validity. We identified any gaps, inconsistencies, or conflicting findings in the literature. Moving on, we synthesized and interpreted the findings from the selected studies, identifying overarching themes, theories, or concepts that emerged. We then discussed how the literature contributed to our understanding of the research objective, linking theory to evidence and evidence to theory to draw broad theoretical conclusions.

Finally, we reported and presented our literature review in a structured and logical manner. This included sections such as introduction, methodology, results, discussion, and conclusion, where we comprehensively summarized our findings.

Inclusion Criteria

The inclusion criteria for selecting studies from 2013 to 2023 were as follows:

Language: First, only studies written in the English language were included to ensure accessibility and readability.

Availability: Then studies that were freely available and had full-text versions were selected to ensure comprehensive access to the complete research articles.

Participants: then studies involving human subjects were included to ensure the applicability of the research findings to the human population.

Study types: Both primary and secondary studies were eligible for inclusion - this encompassed a wide range of study designs, such as randomized controlled trials, systematic reviews, and meta-analyses.

**Figure 2:**
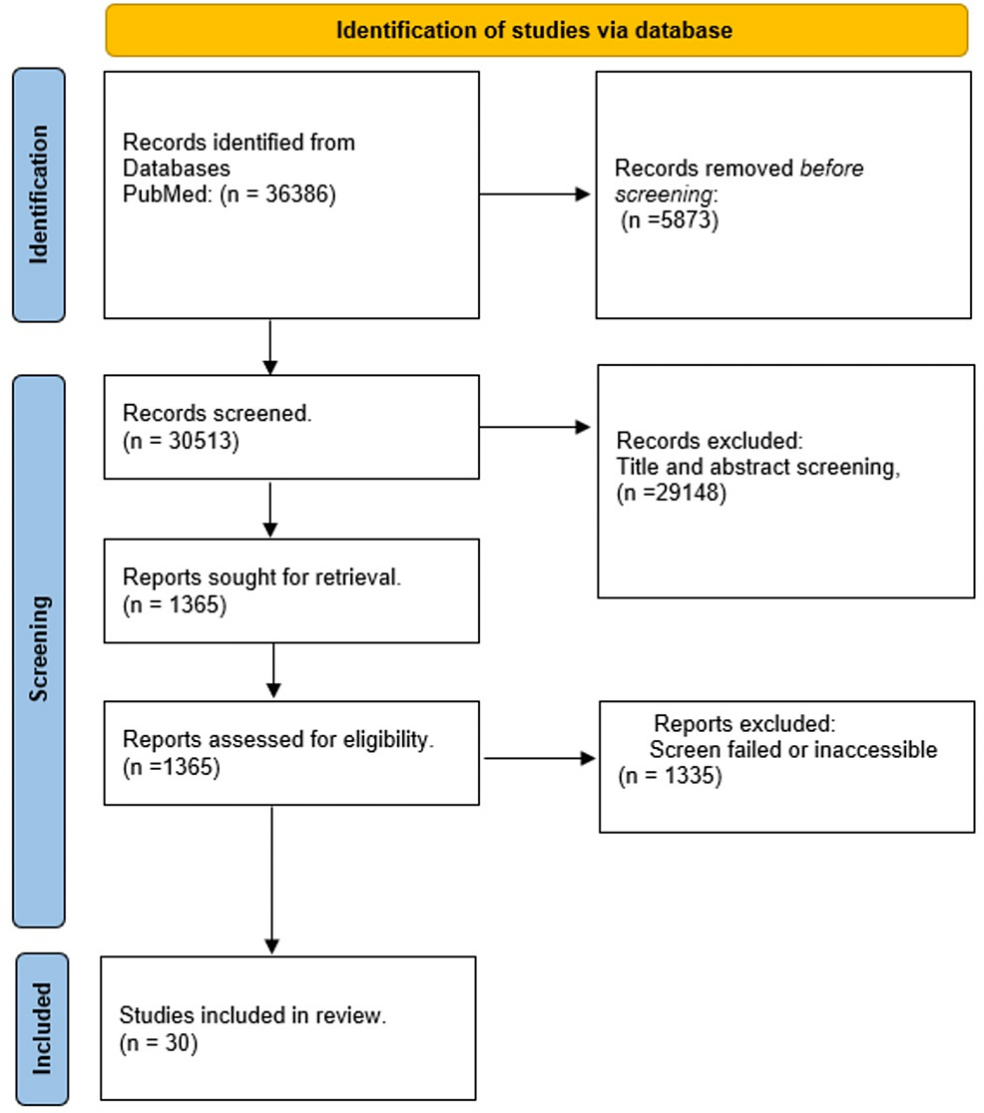
PRISMA flow chart for the selected study

See Figure 2 for the PRISMA flow diagram of included studies (30 studies).

## Review

Some risk factors for bladder cancers

Several risk factors have been associated with the development of bladder cancer, including age, gender, smoking, and occupational exposures [5]. The disease is more common in men than women (four times more likely in men than women). It’s incidence is also observed to increase with advancing age as over 90% of bladder cancer diagnoses are made at age 55 and older [1]. Tobacco smoking is the most important modifiable risk factor accounting for about 50% of all cases in men and 30% of all cases in women [6]. Smokers have been shown to be three times more likely to develop the disease than non-smokers [7]. Occupational exposures to certain chemicals, such as aromatic amines and polycyclic aromatic hydrocarbons, have been linked to an increased risk of bladder cancer accounting for approximately 5-10% of all cases [6]. Other risk factors include chronic bladder infections, family history, genetic predisposition, bladder stones, long term catheterization, and previous radiation therapy to the pelvic area. Chronic bladder infection has been associated with an increased risk of squamous cell carcinoma of the bladder which accounts for less than 2% of all bladder cancer diagnoses. Genetic predisposition may also play a role in the development of bladder cancer, as several genes have been identified that are associated with an increased risk of the disease. Interestingly, some studies suggest that certain dietary factors may have protective effect against bladder cancer including higher intake of fruits and vegetables particularly cruciferous vegetables. Also, physical activity and moderate alcohol consumption have been shown to have a protective effect on bladder cancer risk.

Bladder cancer characteristics and treatment

Bladder cancer is classified into two main categories based on the extent of tumor invasion into the bladder wall: non-muscular invasive bladder cancer (NMIBC), which is limited to the inner lining of the bladder, and muscle-invasive bladder cancer (MIBC), which has spread into the deeper layers of the bladder wall and may also involve surrounding lymph nodes and other organs. NMIBCs, including urothelial cancers, account for the most common type of bladder cancer, making up approximately 70% of all bladder cancer diagnoses, while MIBCs represent the remaining 30% [8]. Other less common types of bladder cancer include squamous cell carcinoma, adenocarcinoma, and small cell carcinoma [7].

The diagnosis of bladder cancer is typically made through a combination of medical history, physical examination, and diagnostic tests, including urine tests, cystoscopy, biopsy, and imaging studies such as CT scans or MRIs. Treatment options for bladder cancer depend on the type and stage of cancer, including surgery, chemotherapy, radiotherapy, targeted therapy, and immunotherapy. NMIBC is usually treated with transurethral resection of the bladder tumor (TURBT), followed by intravesical instillation of Bacillus Calmette-Guerin (BCG) or chemotherapy (such as mitomycin C). MIBC is typically treated with radical cystectomy, which involves the removal of the entire bladder and/or radiation therapy and chemotherapy [1, 7-8]. Neoadjuvant chemotherapy before cystectomy is recommended for MIBC, as it improves survival outcomes. Gemcitabine and cisplatin are the most commonly used chemotherapy agents for MIBC, with a 50-60% response rate. Platinum-based chemotherapy is the standard first-line treatment for advanced or metastatic bladder cancer. Radiotherapy is most commonly used for patients unsuitable for surgery or who decline surgical intervention. It can, however, be used either as primary treatment or as adjuvant therapy following surgery.

Two tremendous breakthroughs in bladder cancer therapy in recent times (2011-2014) were the approval of immune checkpoint inhibitors (ICIs) and the fibroblast growth factor receptor tyrosine kinase inhibitor (FGFR-TKI) Eedafitinib [9]. The approval of FGFR-TKI followed the recent evidence that alterations in the fibroblast growth factor receptor 3 (FGFR3) gene are common in non-invasive bladder cancer and can be used to identify patients who may benefit from targeted therapy [1, 7-9]. Other promising therapeutic targets for bladder cancer include nectin-4, a cell surface protein that is overexpressed in most bladder cancer patients, and enfortumab vedotin, an antibody-drug conjugate that targets nectin-4 [1,3, 4-9]. These recent advances in the understanding of molecular biology and genetics of bladder cancer have improved the diagnosis and treatment of the disease, making targeted therapy and immunotherapy mainstay treatment options in the care of patients with bladder cancer, especially in those with advanced or metastatic disease.

Immune checkpoint inhibitors and bladder cancer

**Figure 3:**
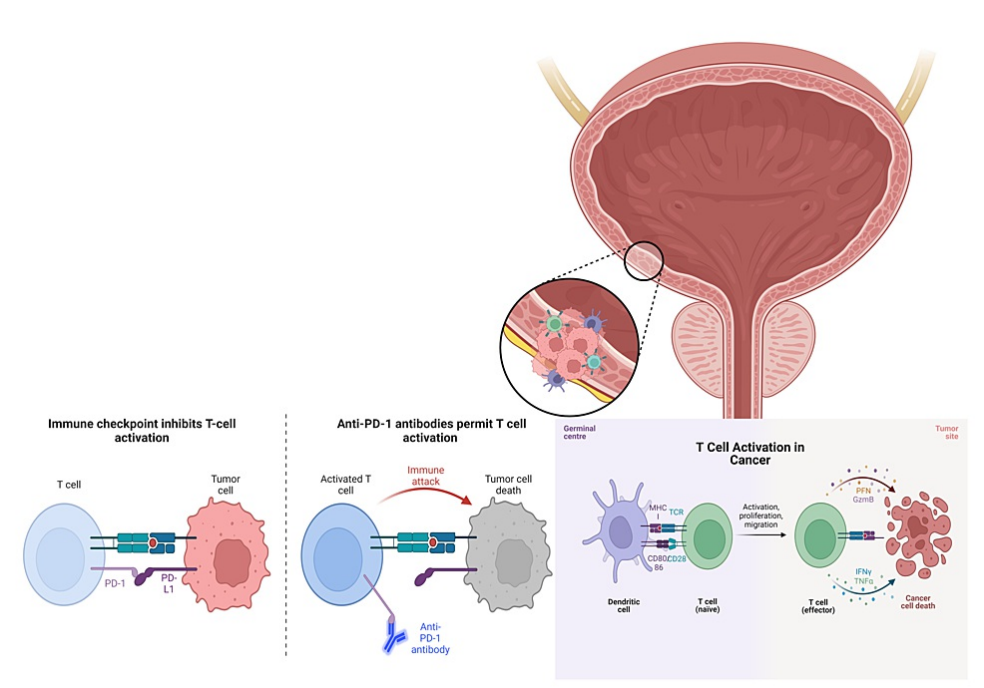
T cell activation PD-1: programmed cell death protein 1 This illustration is an original creation of the authors of this manuscript.

Bladder cancer is a heterogeneous disease, and different types of bladder cancer have different molecular characteristics and clinical outcomes. However, immunotherapy using immune checkpoint inhibitors (ICIs) has revolutionized the treatment of the disease. ICIs block the signaling pathway that inhibits T-cell activity and promotes tumor growth, preventing T-cell exhaustion and allowing the immune system to recognize and attack cancer cells more effectively [4]. The two most targeted checkpoint proteins are PD-1 and CTLA-4. US Food and Drug Administration (FDA) has approved several ICIs, including PD-1 inhibitors (nivolumab, pembrolizumab, and cemiplimab), PD-L1 inhibitors (atezolizumab, durvalumab, and avelumab), and CTLA-4 inhibitor (ipilimumab) [10]. CTLA-4 act early in the immune response to prevent T-cell activation. CTLA-4 inhibitors block the CTLA-4 protein found on T cells allowing them to become activated against cancer cells. On the other hand, PD-1 proteins on T cells bind to PD-L1, often expressed by cancer cells. When PD-1 binds to PD-L1, it sends a signal to the T cell to turn off, preventing the immune system from attacking the cancer cells. By blocking PD-1, PD-1 inhibitors allow the T cell to remain active to continue attacking the cancer cells (Figure 3). Other checkpoint proteins targeted by ICIs include LAG-3, TIM-3, and TIGIT. LAG-3 is found in T cells and other immune cells and can inhibit T-cell activation. TIM-3 is found in T cells and can suppress their activity. TIGIT is found on T cells and natural killer cells and can inhibit their function. Inhibitors targeting these proteins are currently being studied in clinical trials [3, 7-10]. 

ICIs targeting PD-1 and PD-L1 have shown significant clinical efficacy in bladder cancer treatment, especially in patients with high PD-L1 expression [11]. Following the results of the IMvigor210 trial, which showed a durable response rate of 23% in patients with PD-L1 expression ≥ 5% [12], atezolizumab, an anti-PD-L1 antibody, became the first FDA-approved immunotherapy for the treatment of locally advanced or metastatic bladder cancer in 2016 [10-12]. Atezolizumab significantly improved overall survival and progression-free survival in patients with advanced bladder cancer [13], making it a preferred first-line treatment combined with platinum-based chemotherapy for advanced or metastatic bladder cancer. Following subsequent landmark clinical trials, including the KEYNOTE-045 trial [14], and Phase III DANUBE trial [12], four other ICIs have been approved by FDA for bladder cancer treatment, including durvalumab, nivolumab, avelumab, and pembrolizumab [15]. Several ongoing clinical trials are evaluating the efficacy of ICIs in bladder cancer, either as monotherapy or in combination with other treatments, such as chemotherapy or radiation therapy. In a recent phase III trial, the combination of nivolumab and ipilimumab demonstrated a significant improvement in overall survival compared to chemotherapy in patients with previously untreated metastatic urothelial carcinoma [16].

Limitations to the use of ICIs in bladder cancer management

Despite the success of ICIs in bladder cancer management, challenges still exist with their use. A major challenge is identifying biomarkers that can predict the response to treatment. PD-L1 expression has been used as a biomarker to select patients for anti-PD-L1 therapy. However, PD-L1 expression is not a reliable biomarker for patient selection, and many patients with PD-L1-negative tumors still respond to anti-PD-L1 therapy [4]. Also, resistance mechanisms to ICIs have been identified as not all patients respond to therapy. Resistance could be primary if it occurs in tumors with no response to treatment or secondary if it occurs in tumors that develop resistance following the initial response to treatment. The mechanism of resistance to ICIs in bladder cancer is complex and multifaceted, including components of the tumor microenvironment (TME), tumor heterogeneity, and genetic alterations [17-19].

Resistance to ICIs can occur at various stages of T-cell activation, including antigen presentation, T-cell priming, T-cell trafficking, and T-cell effector function. For example, tumors with low mutational burdens or few neoantigens may not effectively activate T-cells, leading to primary resistance. Tumors may also have immune-suppressive factors, such as regulatory T-cells, myeloid-derived suppressor cells, and M2 macrophages, inhibiting T-cell activity and leading to resistance. Additionally, tumors may have defects in interferon signaling or interferon-related gene expression, which are critical for T-cell activation and function, leading to resistance [20]. Also, the inability to present tumor antigens to T cells and the downregulation of major histocompatibility complex (MHC) class I molecules on tumor cells can prevent T-cell recognition and cytotoxicity, leading to resistance to ICIs [21].

Another mechanism of resistance is interferon-gamma signaling. Interferon-gamma (IFN-γ) plays a crucial role in T-cell activation and the upregulation of immune checkpoint molecules such as PD-L1 [22]. However, tumor cells can develop resistance to IFN-γ through mutations in the Janus kinase/signal transducers and activators of transcription (JAK-STAT) pathway, which prevents the upregulation of PD-L1 expression [23-24]. Additionally, the TME can induce the expression of immune checkpoint molecules, such as PD-L1 and IDO, which can suppress T-cell activity [19]. Potential strategies for overcoming ICIs resistance in bladder cancer treatment include combining ICIs with other therapies such as chemotherapies, targeted therapy, or other immunotherapies or using personalized approaches to identify specific biomarkers associated with resistance [24-30].

Managing side effects from these immunotherapeutic agents

Managing the side effects of immunotherapy involves following guidelines established by reputable organizations. The National Comprehensive Cancer Network (NCCN) provides guidelines for the management of immunotherapy-related adverse events in the United States. The NCCN emphasizes the importance of promptly recognizing and grading the severity of side effects using established grading systems such as the Common Terminology Criteria for Adverse Events (CTCAE) [31]. Patient education is crucial, ensuring individuals and caregivers are aware of potential side effects, their symptoms, and when to seek medical attention. Symptom management involves supportive care measures such as analgesics, antipyretics, and antiemetics. Additional interventions, such as corticosteroids or immunosuppressive agents, may be necessary depending on the specific side effect.

Close collaboration between oncologists and specialists from various fields is essential to manage side effects in specific organ systems effectively [32]. The European Society for Medical Oncology (ESMO) provides guidelines on managing toxicities related to immune checkpoint inhibitors in Europe. These guidelines stress early recognition and grading of side effects, utilizing validated grading systems. A multidisciplinary approach is encouraged, involving specialists like dermatologists, pneumologists, endocrinologists, and gastroenterologists to address specific toxicities. Symptom management is tailored to the side effect and its severity, incorporating supportive care measures, corticosteroids, or immunosuppressive agents as needed. In severe cases of immune-related adverse events, temporary or permanent discontinuation of immunotherapy may be required, and the decision to rechallenge should be made based on the severity of the event and potential treatment benefits [33]. It is important to note that medical guidelines are subject to updates.

Specifically, treatment-related adverse events(TRAEs) and immune-related adverse events (irAEs) are another major concern with immunotherapy [24]. By acting as “off switches” to the immune system, ICIs inhibitors could over-activate the immune system resulting in significant irAEs that could potentially be life-threatening. These irAEs could result in multiple organ system toxicities, including dermatological, gastrointestinal, hepatic, endocrine, pulmonary, and neurological toxicities. ICIs treatment in bladder cancer was associated with a higher incidence of irAEs than chemotherapy, with the most common irAEs being dermatological, gastrointestinal, and hepatic toxicities [25]. Systematic review and meta-analysis of randomized controlled trials of ICIs in patients with bladder cancer found that ICIs were associated with a higher incidence of TRAEs than chemotherapy, but the incidence of grade 3-5 TRAEs was similar between the two groups. The most common TRAEs associated with ICIs in bladder cancer were fatigue, pruritus, and nausea, while the most common grade 3-5 TRAEs were diarrhea, fatigue, and colitis [1,4,6,9,13,16,24-26]. Further research is needed to optimize the use of ICIs to improve patient outcomes.

Future directions for research on ICIs for bladder cancer

The introduction of ICIs in bladder cancer management has shown promising results, especially in patients with advanced or metastatic disease who have failed previous therapies. The ICI therapy has emerged as a promising treatment approach for various cancers. However, in some cases, patients may experience what is referred to as "immune checkpoint inhibitor failed therapy." This term indicates that the treatment with immune checkpoint inhibitors did not achieve the desired clinical outcomes or resulted in disease progression [26-34]. Available guidelines typically define immune checkpoint inhibitor failed therapy based on specific criteria. These criteria may include progression of the disease, lack of response, treatment intolerance or adverse events, and disease recurrence after an initial response [35]. The progression of the disease is determined by assessing the growth or spread of cancer through imaging techniques, such as CT scans or MRIs, or by evaluating the patient's symptoms [36-39]. A lack of response can be established by assessing tumor size, biochemical markers, or other indicators of disease activity.

Some patients may also experience treatment intolerance or adverse events that prevent the continuation of ICI therapy. These side effects may include immune-related toxicities affecting various organs or systems in the body. Some patients may initially respond to ICIs and experience tumor regression. However, over time, the disease may progress again, indicating a loss of response or development of resistance to the therapy. It's important to note that the specific guidelines and criteria for defining ICI failed therapy may vary depending on the type of cancer being treated and the specific ICIs used. These guidelines are typically established by medical societies, regulatory agencies, and expert consensus based on clinical trial data and real-world experience. Medical oncologists and healthcare providers rely on these guidelines to assess treatment response and guide subsequent therapeutic decisions for patients who have not benefited from ICI therapy. By identifying patients who have experienced ICI failed therapy, alternative treatment options can be explored to optimize patient outcomes [34-39].

However, the response rates to ICIs can vary among patients, and not all bladder cancer types or patients with noninvasive or low-grade tumors may benefit equally from ICIs. Therefore, continued research is crucial to optimizing their use in bladder cancer. Several strategies are currently being evaluated and would potentially become the future of immune therapy in bladder cancer management. One potential strategy is combining ICIs with other immune modulators, such as cytokines, toll-like receptor agonists, checkpoint inhibitors, or other chemo or radiotherapy modalities [27]. Another area of research is the identification of potential biomarkers other than PD-L1, such as tumor mutation burden, gut microbiome, and microsatellite instability that can predict the response to ICIs in bladder cancer [28].

Several novel immune-based therapies are also currently under investigation for the treatment of bladder cancer. These include adoptive cell therapy (ACT) and cancer vaccines [29]. ACT is a promising immune-based therapy that involves the infusion of autologous T cells that have been genetically engineered to express a chimeric antigen receptor (CAR) specific to a tumor antigen. ACT has shown remarkable efficacy in treating some types of cancer, such as leukemia, melanoma, and lymphoma. In bladder cancer, several preclinical and clinical studies have demonstrated the feasibility and safety of ACT. Combining ACT with ICIs may enhance the efficacy of both treatments by promoting T-cell function and overcoming resistance mechanisms. Cancer vaccines aim to stimulate the immune response against tumor antigens by presenting them to the immune system. They have been used for many decades and have shown relatively modest clinical activity across cancers, including bladder cancer. Novel strategies targeting neoantigens, such as personalized vaccines, may improve clinical outcomes in bladder cancer [29]. Additionally, combinations of ICIs with adoptive cell therapy and cancer vaccines are being investigated and may further improve prognosis in bladder cancer [30].

## Conclusions

In conclusion, immune checkpoint inhibitors have transformed the treatment landscape of several types of cancer, including bladder cancer. ICIs targeting PD-1, PD-L1, and CTLA-4 have shown significant clinical efficacy in treating advanced bladder cancer. However, identifying reliable biomarkers to predict the response to ICIs remains challenging. Further research is needed to better understand their mechanism of action, develop new combinations with other cancer treatments, identify novel predictive biomarkers, and improve patient selection for ICIs therapy in bladder cancer.
